# Effect of dietary supplementation of *Bacillus subtilis* TLRI 211-1 on laying performance, egg quality and blood characteristics of Leghorn layers

**DOI:** 10.5713/ab.22.0274

**Published:** 2023-01-08

**Authors:** Ming-Yang Tsai, Bor-Ling Shih, Ren-Bao Liaw, Wen-Tsen Chen, Tsung-Yu Lee, Hsi-Wen Hung, Kuo-Hsiang Hung, Yih-Fwu Lin

**Affiliations:** 1 Animal Industry Division, Livestock Research Institute (LRI), Council of Agriculture (COA), Hsinhua, Tainan 712009, Taiwan; 2 Graduate Institute of Bioresources, National Pingtung University of Science and Technology, Neipu, Pingtung 91201, Taiwan; 3 Nutrition Division, Livestock Research Institute (LRI), Council of Agriculture (COA), Hsinhua, Tainan, 712009, Taiwan; 4 Physiology Division, Livestock Research Institute (LRI), Council of Agriculture (COA), Hsinhua, Tainan, 712009, Taiwan

**Keywords:** *Bacillus subtilis*, Egg Quality, Egg Production Performance, Leghorn Layer

## Abstract

**Objective:**

TLRI 211-1 is a novel *Bacillus subtilis* strain. This experiment was to investigate dietary supplementation of TLRI 211-1 on laying performance, egg quality and blood characteristics of layers.

**Methods:**

One hundred and twenty 65-wk-old Leghorn layers were divided into four treatment groups for 8 weeks experiment. Each treatment had three replicates. The basal diet was formulated as control group with crude protein 17% and metabolizable energy 2,850 kcal/kg and supplemented with TLRI 211-1 0.1%, 0.3%, and commercial *Bacillus amyloliquefaciens* 0.1% as treatment 2, 3 and 4 groups, respectively. Both TLRI 211-1 and commercial *Bacillus amyloliquefaciens* were adjusted to contain 1×10^9^ colony-forming unit (CFU)/mL (g), hence the 0.1% supplemental level was 1×10^9^ CFU/kg.

**Results:**

The results showed that TLRI 211-1 0.3% and commercial *B. amyloliquefaciens* groups had higher weight gain than the other groups; TLRI 211-1 0.1% group had better feed to eggs conversion ratio than the control and commercial *B. amyloliquefaciens* groups (p<0.05). *Bacillus subtilis* supplementation increased yolk weight (p<0.05). In egg quality during storage, TLRI 211-1 0.1% had higher breaking strength than the control group at the second week of storage (p<0.05). At the third week of storage, TLRI 211-1 0.3% had higher Haugh unit (p<0.05). Hens fed diets supplemented with TLRI 211-1 0.3% significantly decreased blood triglyceride levels and increased blood calcium levels (p< 0.05). TLRI 211-1 0.3% group had lower H_2_S (p<0.05) and hence had less unpleasant odor in excreta of hens.

**Conclusion:**

In conclusion, supplementation with 0.1% TLRI 211-1 can significantly improve feed to eggs conversion ratio. TLRI 211-1 supplementation also can maintain eggs at their optimum quality level during storage. The study showed that *B. subtilis* TLRI 211-1 can be used as feed additives for improving egg production performance and egg quality.

## INTRODUCTION

Probiotic feed additives are recently commonly applied in livestock diets to promote health, growth, and suppression of environmental odors. *Bacillus subtilis* (*B. subtilis*) is one of the bacteria permitted to be used as feed additive in poultry diet by the Council of Agriculture, Taiwan [1]. It has the characteristics of fast growth, resistance to acid, alkaline and heat, and endospore formation. Endospores can reach the gut to grow and reproduce withstanding the pelleting process of feed and strong acid environment in stomach. It is beneficial to the balance of gut flora, promotion of immune function, disease resistance and growth. Beneficial properties of *Bacillus* probiotics can be used as feed additives to improve feed efficiency and survival rate of farm animals [2,3]. The Food and Agriculture Organization of the United Nations indicated that boosting efficiency of livestock production and resource use is one of the five practical actions towards low-carbon livestock production [4]. Hence, *Bacillus* probiotics may also play a role in decreasing carbon emissions of livestock production. Furthermore, achieving low-carbon livestock production is an important goal for maintaining a sustainable environment.

In research on growing-finishing pigs, Upadhaya et al [5] indicated that diets supplemented with *Bacillus*-based feed additive improved growth performance, increased nutrient digestibility, and decreased fecal ammonium emission. Kaewtapee et al [6] showed that dietary supplementation of *B. subtilis* and *Bacillus licheniformis* results in abundant and healthy bacteria in the gut and improved health and performance of growing pigs.

In poultry research, a previous study showed that broilers fed with newly developed *B. subtilis* HB2 as feed additive had better growth performance, less mortality and ammonium emission [7]. Study on the effects of dietary probiotic, prebiotic and symbiotic supplementation of broilers indicated that all can improve growth performance without affecting blood physiological value and carcass mass, and the broilers have carcass fat content than the control group under restricted feeding [8]. Hatab et al [9] and Lei et al [10] showed that feeding laying hens with *B. subtilis* can significantly improve egg production performance and quality. Other studies pointed out that *B. subtilis* can adjust the gut probiotic ecology of layer hens, which significantly increased the number of probiotic bacteria in excreta and cecum under long-term feeding and can maintain the healthy state of the gut [11,12].

The *B. subtilis* TLRI 211-1 (TLRI 211-1) was screened, identified, and purified by Taiwan Livestock Research Institute (TLRI; Hsinhua, Tainan, Taiwan). It had high activities of proteases, lipases, α-amylases, xylanases, β-glucanases and phytases. It also had the characteristics of acid and bile salt resistance, and no urease activity was found [13]. In the experiment evaluating litter ammonia content of broilers, supplementation of 0.1% TLRI 211-1 lowered litter ammonia content compared to the control group during middle to late rearing period [13]. Research of TLRI showed that goat kids’ diets added with TLRI 211-1 decreased 15% feces ammonia content, and air quality of goat house was improved when the bacteria count of diet reached 1×10^8^ colony-forming unit (CFU)/kg [14]. Lee et al [3] also indicated that dietary supplementation of TLRI 211-1 can significantly improve the weight gain, growth efficiency and carcass weight, and decreased abdominal fat of broilers when the bacteria count of diet reached 1×10^8^ CFU/kg. Hence, TLRI 211-1 strains can be used as probiotic feed additives to promote the growth performance of chickens.

Since the gut probiotic bacteria flora is beneficial to the absorption of protein in the feed, it reduces the excretion of ammonia and can reduce the number of harmful bacteria or pathogenic bacteria in the gut and excreta. Dietary supplementation with prebiotics, probiotics, and phytogenic substances was found to affect gut microbiota quality and the improvement in intestinal epithelial barrier might be used to reduce the usage of antibiotics in poultry farming [2]. Furthermore, a combination of acetic acid and prebiotic was also found to decrease the shedding of Salmonella in feces and thus reducing the health threat [15]. The objective of this experiment was to investigate the effects of novel probiotic *B. subtilis* TLRI 211-1 on the egg production performance, egg quality and blood characteristics of Leghorn layers.

## MATERIALS AND METHODS

The trial was conducted in the experimental chicken barn in TLRI at Hsinhua, Tainan, Taiwan. All experimental procedures were approved by the Institutional Animal Care and Use Committee (IACUC No. 110-25) of TLRI.

### Preparation of *B. subtilis* TLRI 211-1

*Bacillus subtilis* of TLRI 211-1 was screened from the activated sludge at TLRI. After species identification, high temperature resistance (50°C), and spore-producing ability test, the *B. subtilis* was selected and inoculated in Tryptic Soy Broth culture media and placed in a 30°C incubator for 24 hours. After a process of adjusting with food grade silicon dioxide, mixing well, centrifuging to remove the upper liquid and air drying in a 40°C oven, the total content of viable sporulated TLRI 211-1 bacteria used in this experiment was 1×10^9^ CFU/g. Commercial *B. amyloliquefaciens* (CML. *B. amyloliquefaciens*) used in the treatment 4 was provided by Yungstrong, Vetnostrum Animal Health Co., Ltd, (Hsinchu, Taiwan) and adjusted to the content of 1×10^9^ CFU/g.

### Experimental animals and treatment

A total of one hundred and twenty Leghorn layers (Hy-line W-36) at 65 weeks of age were divided into four treatment groups. Each bird was housed in an individual cage measuring 23 cm×42 cm×40 cm (width×length×height) in an open-sided house. Each treatment group, containing 30 birds, was equally separated into three experimental units (replicates). Feed and water were provided *ad libitum* with a light regimen of 16 h of continuous light (provided 10 to 20 Lux artificial light) per day in open-sided housing. The experiment was conducted for 8 weeks. The corn soybean basal diets (mash form) for each group were formulated consulting Hy-Line Management Guides [16] and NRC [17] to contain 17.0% crude protein, 2,850 kcal/kg metabolizable energy, 4.15% calcium and non-phytate phosphorus 0.26% (Table 1). The TLRI 211-1 was added 0.0%, 0.1% and 0.3% for the control, treatment 2 and treatment 3 groups, respectively. Treatment 4 was added 0.1% CML. *B. amyloliquefaciens*. The hens did not receive any antibiotics during the entire experimental period from 65 to 73 weeks of age.

### Measurements

- Body weight was measured at the beginning and end of the experiment to determine the weight change of hens.
- Egg weight was measured two days a week and feed consumption was recorded every week. The number of eggs produced, and abnormal egg count were recorded every day. The hen-day egg production, egg mass and feed conversion ratio were calculated as follows:
-
$$\begin{array}{l} \text{Hey}-\text{day production }(\%)=(\text{total number of eggs produced}/\text{number of hens}/\text{days of egg production})\times 100\\ \text{Daily egg mass per hen }(\text{g}/\text{d})=(\text{number of eggs produced}\times \text{average egg weight})/\text{days of egg production}\\ \text{Feed conversion ratio}=\text{feed intake per hen per day}/\text{egg mass per hen per day} \end{array}$$
- Egg quality: Twelve eggs were collected per group every four weeks for measurement of eggshell quality, egg quality and Haugh unit using Digital Egg Tester, DET6500 (Nabel Co., Ltd. Kyoto, Japan).
  - Eggshell quality determination included eggshell strength, eggshell thickness, eggshell weight, and eggshell strength. Egg quality included yolk percentage, yolk color, yolk height, yolk diameter and albumen height.
  - Eggshell thickness determination: A piece of eggshell at the blunt end, tip and equatorial part of the egg were taken and measured using micro measuring instrument to a decimal point of 3 digits. The average of the three measurements was the thickness of eggshell [18].
  - After the eggs were weighed, the eggshell, yolk and albumen weight were taken separately, and the eggshell weight, yolk weight and albumen weight were calculated as the percentage of egg weight.
- Blood characteristics: Eight hens from each group were randomly selected for blood test every four weeks. Five mL of blood samples were taken from the wing vein. Serum was separated by the Centrifugal Separator (1,700×g, 15 min) and stored in a −20°C freezer for analysis. Immunoglobulin (IgA, IgG, and IgM) were determined using chicken ELISA quantitation set (Bethyl Laboratories Inc., Montgomery, TX, USA). Blood glucose, bilirubin, creatinine, uric acid, urea nitrogen, creatine phosphatase, glutamate oxaloacetate transaminase (GOT), pyruvate transaminase (GPT), total protein, albumen, alkaline phosphatase, total cholesterol, triglycerides, high density lipoprotein (HDL), low density lipoprotein (LDL), inorganic phosphorus, and amylase activity were analyzed using automatic analyzer (Hitachi 7176A; Tokyo, Japan) [19].
- Determination of egg quality during egg storage: At the end of the experiment, 48 eggs were selected from each treatment group and stored at room temperature (23°C to 30°C) for 4 weeks. Twelve eggs from each group after 2, 3, and 4 weeks of storage were selected for the determination of egg quality and Haugh unit as the above egg quality determination method.
- Determination of the odor of laying hen excreta: At the end of the experiment, 2 replicates of fresh feces were taken from each group, 10 hens per replicate. Each excreta sample was mixed well and 5 g were placed in a 500 mL Erlenmeyer flask, and the bottle sealed with a parafilm for 5 min. One hundred mL of gas was suctioned with a gas sampler (Kitagawa AP-20) from 5 cm above the bottom of the bottle and poured into the ammonia detection tube (Kitagawa Gas Detector Tube Ammonia 105SC or 105SD) for determination of ammonia concentration. Each sample was conducted 3 replicate tests.
-
$$\begin{array}{l} \text{Calibration formula:}\text{ Measured concentration }(\text{mg}/{\text{m}}^{3})\\ =\text{measured concentration }(\text{ppm})\times (17.03/22.4)\times (273/273+\text{t}), \end{array}$$
- Approximate nutrient contents of experimental diets: Diet samples were sent and analyzed at the Feed Analysis Center, Animal Nutrition Division, TLRI using AOAC [20] methods.

### Statistics analysis

Trait data from the experiment was analyzed using the SAS 9.4 (SAS Institute Inc., Cary, NC, USA) with general linear model Procedure for variant analysis. For the statistical analysis of feed intake, feed to eggs conversion rate, egg mass and laying rate, average performance of every replicate with 10 birds was treated as an experimental unit. Before analysis, all percentage data were transformed to arcsine of the square root to normalize data distribution. Because no statistical differences were observed between the transformed and not transformed, the statistics shown in this paper were from the untransformed data. If there was significant difference, the least squares mean was adopted for difference analysis among groups. The significant difference level was set at p<0.05.

## RESULTS AND DISCUSSION

### Body weight changes and excreta odor

Effects of dietary supplementation of *B. subtillis* and *B. amyloliquefaciens* on body weight changes and excreta odor of laying hens are shown in Table 2. During the experimental period in the layer house, the average temperature was 26.5°C (21.6°C to 34.5°C) and the relative humidity was 48% to 88%. In the end of the experiment, the body weight and weight gain of layers in the control group were lower than those in TLRI 211-1 0.3% and CML. *B. amyloliquefaciens* 0.1% groups (p<0.05). It indicated that the supplementation of *Bacillus* spp. may promote nutrient absorption in chickens. The results agreed with the research of Leeson and Summers [21] who showed that the intake of *B. subtilis* ferment by layer hens can increase the weight of chickens during growth and egg laying period.

The odor composition in ammonia (NH_3_) and methylamine (CH_3_NH_2_) of layer excreta was not affected by the addition of *B. subtilis* and *B. amyloliquefaciens*. The odor composition in hydrogen sulfide (H_2_S) was lower in TLRI 211-1 0.3% and CML. *B. amyloliquefaciens* groups than that the control group (p<0.05). It indicated that TLRI 211-1 had the effect of significantly reducing odor. Ding et al [22] research showed that layer diets supplemented with *Bacillus* spp., yucca, and *B. subtilis* can reduce ammonia concentration in excreta. In this experiment, although the ammonia odor was not significantly reduced in TLRI 211-1 0.3% group, but hydrogen sulfide odor was significantly reduced. This is like the above conclusion that the addition of *B. subtilis* may reduce excreta odor.

### Egg production performance

Effects of dietary supplementation of *B. subtillis* and *B. amyloliquefaciens* on feed intake and egg production performance of laying hens are shown in Table 3. There was no difference in feed intake, egg production, egg mass and soft-shell egg rate among groups during 8 weeks of experimental period. For feed conversion ratio, TLRI 211-1 0.1% group was better than the control and CML. *B. amyloliquefaciens* group (p< 0.05).

Although no statistical difference was found, dietary supplementation of TLRI 211-1 0.1% had the highest hen-day egg production and egg mass. The results showed that the TLRI 211-1 may induce digestive enzyme activity, which helps nutrient absorption and thus improves the feed conversion rate.

Guo et al [23] in a long-term experiment reported that Hy-Line brown layers fed with 10^5^ to 10^8^ CFU/kg *B. subtillis* CGMCC 1.921 for 24 weeks can improve feed conversion rate, but the feed intake, egg production rate and egg weight were not affected. In addition, egg quality was improved via the reduction of fecal *E. coli* and beneficial modulation of cecal microbiota. Research of Forte et al [12] showed that Hy-Line layer hens fed with 0.05% *B. subtilis* for 14 weeks had increased blood estrogens and reduced blood inflammation indicators such as interleukin-1 and tumor necrosis factor-α concentrations, which verified that *B. subtilis* can be used in reducing infection and bone destruction, and maintaining good health, less fractures, and welfare of laying hens. In an aging layer hen experiment, *B. subtilis* was found to improve feed conversion rates in layer hens aged 72 to 79 weeks and eggshell quality can be significantly improved accompanying 4% dietary calcium content [24]. The laying hens in this experiment were aged 65 to 73 weeks. The above results may verify part of the reason why the consumption of *B. subtilis* improved the feed conversion rate of eggs.

### Egg quality

Effects of dietary supplementation of *B. subtillis* and *B. amyloliquefaciens* on egg quality of laying hens are shown in Table 4. In egg quality, there was no significant difference on egg weight, eggshell strength, shell weight, yolk weight percentage, eggshell thickness, albumen height, yolk redness (a value), yellowness (b value) and Haugh at the 4th week of the experiment. During the 5th to 8th weeks of the experimental period, CML. *B. subtilis* group had lower albumen weight than that of the control group (p<0.05). There was no significant difference among the four groups in egg weight, eggshell traits, albumen height, Haugh unit, yolk traits and yolk color. For the total experimental period from 65 to 73 weeks of age, it showed that CML. *B. subtilis* groups had significantly higher yolk weight and lower albumen than the control group (p<0.05). The TLRI 211-1 group also had higher yolk weight than the control group (p<0.05). The other quality traits such as egg weight, eggshell traits, yolk traits, yolk color, albumen height, and Haugh unit were similar among the groups.

Zou et al [25] used Lohmann layer hens (48-week-old) fed with *B. subtilis* for 10 weeks indicating that *B. subtilis* could be used as a health promotor to reduce overproduction-induced inflammation and associated bone damage and to increase marketable egg production. The skeletal system is an important source of minerals for eggshell formation. Attia et al [26] also found that increasing dietary Ca levels to 4% during the late production phase could improve laying performance. Further studies are needed to identify whether age of hens may effect eggshell quality.

### Egg quality during storage

Effects of dietary supplementation of *B. subtillis* and *B. amyloliquefaciens* on egg quality during four-week storage in room temperature are shown in Table 5. When eggs were stored for 2 weeks, the eggshells’ breaking strength was significantly higher in TLRI 211-1 0.1% group than that in the control group (p<0.05). The TLRI 211-1 0.3% and CML. *B. amyloliquefaciens* 0.1% groups had significantly higher eggshell weight ratio than the control group (p<0.05). The other characteristics of egg quality had no significant difference among the groups. When eggs were stored for 3 weeks, the albumen height and yolk color in TLRI 211-1 0.1% and 0.3% groups were significantly higher than those in the control and CML. *B. amyloliquefaciens* 0.1% group. TLRI 211-1 0.3% group also had significantly higher Haugh unit (p<0.05). TLRI 211-1 0.1% group had significantly higher yolk weight percentage (p<0.05). It was significantly higher in the yolk diameter of TLRI211-1 0.1% and CML. *B. amyloliquefaciens* groups (p<0.05), while the other egg quality items, including egg weight, albumen weight and other yolk traits, had no significant difference among the groups. When eggs were stored for 4 weeks, the control group showed significantly lower egg Haugh unit (p<0.05), and the TLRI 211-1 0.3% and the CML. *B. amyloliquefaciens* groups maintained higher freshness. The TLRI 211-1 0.3% group also had higher albumen weight and darker yolk color (p<0.05) and higher egg breaking strength (p>0.05). The other egg quality was not affected by the addition of *B. subtilis* or *B. amyloliquefaciens*.

Guo et al [23] reported that intake of *B. subtilis* increased eggshells strength of Hy-Line layers. The authors speculated that probiotics could maintain a lower pH environment and improve the absorption of calcium and phosphorus in the intestine. It has also been found to improve the apparent digestibility of calcium and phosphorus in ileum of broilers [27]. Zou et al [25] showed that supplementation of probiotic lactic acid or *B. subtilis* can improve the activity of lysozyme in eggs, which that lysozyme has antibacterial properties.

The storage of eggs for 4 weeks in this test may help maintain Haugh unit due to higher eggshell quality or lysozyme activity. The results indicated that dietary supplementation of *B. subtilis* may have the effect of maintaining egg freshness at room temperature.

### Blood characteristics

Effects of dietary supplementation of *B. subtillis* and *B. amyloliquefaciens* on blood characteristics of laying hens are shown in Table 6. The average of blood characteristics at week 4 and 8 (n = 16) showed that blood glucose, total cholesterol, HDL, LDL, uric acid, creatinine, amylase, urea nitrogen, total protein, total bilirubin, albumin, globulin and albumin/globulin ratio, GOT, GPT, alkaline phosphatase and creatine phosphatase content or activity were not affected by the treatments. Similar result was found in the research of FAO [4] who fed broilers with TLRI 211-1.

In addition to the above blood characteristics, TLRI 211-1 0.3% group significantly reduced triglyceride content (p<0.05). TLRI 211-1 0.1% and 0.3% groups significantly increased sodium contents; TLRI 211-1 0.3% and CML. *B. amyloliquefaciens* groups significantly increased calcium contents (p< 0.05). A report showed that layer hens fed *B. subtilis* with high calcium level could increase eggshell and blood calcium contents, enhance intestinal calcium absorption and small intestinal villus height; relative calbindin-D28k (CALB1) mRNA level of laying hens in the late phase of production was also increased [24]. Fathi et al [28] found that layer hens fed with 200 to 400 mg/kg of *B. subtilis* during the hot season had significantly lower blood cholesterol and triglycerides, which was consistent with the results of this experiment on the reduction of blood lipid content by supplementing TLRI 211-1.

Zou et al [25] indicated that layer hens fed with *B. subtilis* had lower blood phosphorous and higher femur magnesium concentrations. In this study laying hens fed with *B. subtilis* tended to reduce GOT. GOT is an enzyme that is normally present in liver and heart cells. A high level of GOT released into the blood may be a sign of liver or heart damage.

### Blood antibody titers

Effects of dietary supplementation of *B. subtillis* and *B. amyloliquefaciens* on blood immunoglobulin of laying hens are shown in Table 7. The TLRI 211-1 0.3% and the CML. *B. subtilis* groups had significantly higher blood IgA levels than the other groups at the 4th week (p<0.05). There was no significant difference in IgM and IgG levels among the groups. However, at the 8th week and the average of whole experimental period, the content of immunoglobulins such as IgA, IgM, and IgG in the blood of laying hens were not affected by the addition of *B. subtilis* or *B. amyloliquefaciens* to the feed.

Research of Fathi et al [28] showed that laying hens fed with 400 ppm *B. Subtilis* can improve the cellular immune response (PHA-P); 200 and 400 ppm *B. subtilis* can increase the IgM content in the blood, whereas the IgA and IgY levels were not affected. Mountzouris et al [29] in broilers indicated that different levels of probiotic had no difference on plasma immunoglobulin between treatments. Zhang et al [30] used several combinations of probiotics in diets indicating a positive impact on immune response of layer hens and suggested that probiotics could enhance the immune system. Further studies are needed to investigate the influence of age and environmental temperature on the immune response.

## CONCLUSION

We conclude that laying hens fed with *B. subtillis* TLRI 211-1 0.1% tend to increase egg production rate and can significantly improve the feed conversion rate. Incremental TLRI 211-1 supplementation up to 0.3% can had higher Haugh unit and maintain longer freshness during storage. For egg production performance and egg quality, *B. subtillis* TLRI 211-1 is better than CML. *B. amyloliquefaciens* products in this experiment. It has the potential of becoming probiotic feed additives for layer hens.

## Figures and Tables

**Table 1:** Diet formula and compositions offered to Leghorn laying hens in Bacillus subtilis TLRI 211-1 experiment

Items	
Ingredient (%)	
Corn, ground	53.10
Soybean meal, CP 43%	27.00
Fish meal, CP 65%	3.00
Soybean oil	3.50
Wheat bran	-
Dicalcium phosphate	1.60
Limestone, pulverized	10.70
Salt	0.30
Choline chloride, 50%	0.20
DL-methionine	0.20
Vitamin-mineral premix^1)^	0.40
Total	100.00
Calculated value (%)	
Crude protein	17.03
ME (kcal/kg)	2,850
Calcium	4.15
Total phosphorus	0.51
Nonphyate phosphorus	0.26
Total	100.00
Analyzed value (%)	
Crude protein	17.81
Calcium	4.21
Total phosphorus	0.68

CP, crude protein; ME, metabolizable energy.

1) Vitamin premix supplied per kilogram of diet: vitamin A, 3,000 IU; vitamin D_3_, 400 IU; vitamin E, 10 IU; vitamin K_3_, 1 mg; vitamin B_1_, 3.6 mg; vitamin B_2_, 5.4 mg; vitamin B_6_, 7.0 mg; Ca-pantothenate, 20.0 mg; niacin, 70 mg; biotin, 0.3 mg; folic acid, 1.1 mg; vitamin B_12_, 0.02 mg, Mineral premix supplied per kilogram of diet: Cu (CuSO_4_·5H_2_O, 25.45% Cu), 8 mg; Fe (FeSO_4_·7H_2_O, 20.09% Fe), 80 mg; Mn (MnSO_4_·H_2_O, 32.49% Mn), 60 mg; Zn (ZnO, 80.35% Zn), 40 mg; Se (NaSeO_3_, 45.56% Se), 0.15 mg.

**Table 2:** Effects of dietary supplementation of *Bacillus subtillis* and *B. amyloliquefaciens* on body weight changes and excreta odor of Leghorn laying hens

Items	Control	TLRI 211-1		CML. additive 0.1%	SEM	p-value

		0.1%	0.3%			
BW at start (g/hen)	1,431	1,452	1,491	1,454	23.7	0.34
BW at end (g/hen)	1,456^b^	1,470^b^	1,541^a^	1,498^a^	30.9	<0.05
Gain (g/hen)	24.1^b^	18.4^b^	50.8^a^	43.9^a^	19.1	<0.05
Odor of excreta (n = 20)	---------------------------------------------------------- mg/m^3^ --------------------------------------------------					
Ammonia (NH_3_)	9.89	10.6	4.53	12.0	5.13	0.69
Hydrogen sulfide (H_2_S)	7.00^a^	4.34^ab^	2.89^b^	1.95^b^	1.38	<0.05
Methylamine (CH_3_NH_2_)	9.17	9.64	3.32	10.9	4.08	0.42

CML. Additive: commercial *B. amyloliquefaciens* product; SEM, standard error of the mean; BW, body weight.

a,b Means with different letters within the same row are significantly different at 5% level (p<0.05).

**Table 3:** Effects of dietary supplementation of *Bacillus subtillis* and *B. amyloliquefaciens* on feed intake and egg production performance of Leghorn laying hens

Items	Control	TLRI 211-1		CML. additive 0.1%	SEM	p-value

		0.1%	0.3%			
0 to 4 wk						
Feed intake (g/bird/d)	101	98	100	98	1.27	0.19
Hen-day egg production (%)	91.6	93.0	89.4	88.6	1.68	0.22
Egg mass (g/d/hen)	57.8	58.4	57.7	55.4	1.52	0.47
Feed conversion rate (feed intake/egg mass)	1.76^a^	1.67^b^	1.73^ab^	1.79^a^	0.02	<0.05
Broken and soft-shelled eggs (%)	0	0	0.13	0.31	0.13	0.18
5 to 8 wk						
Feed intake (g/bird/d)	100	99	103	102	2.03	0.68
Hen-day egg production (%)	89.9	93.2	90.4	92.3	1.40	0.35
Egg mass, g/d/hen	54.5	56.9	56.1	56.4	1.33	0.61
Feed conversion rate (feed intake/egg mass)	1.83	1.74	1.82	1.80	0.03	0.09
Broken and soft-shelled eggs (%)	0.18	0.12	0.21	0.26	0.17	0.89
0 to 8 wk						
Feed intake (g/bird/d)	101	98	101	100	1.62	0.63
Hen-day egg production (%)	90.1	92.9	90.7	90.3	1.35	0.38
Egg mass (g/d/hen)	56.3	58.2	57.3	56.3	1.38	0.79
Feed conversion rate (feed intake/egg mass)	1.79^a^	1.70^b^	1.76^ab^	1.78^a^	0.02	<0.05
Broken and soft-shelled eggs (%)	0.07	0.06	0.17	0.28	0.11	0.34

CML. Additive: commercial *B. amyloliquefaciens* product; SEM, standard error of the mean.

a,b Means in the same row with different letters are significantly different at 5% level (p<0.05).

**Table 4:** Effects of dietary supplementation of *Bacillus subtillis* and *B. amyloliquefaciens* on egg quality of Leghorn laying hens

Items	Control	TLRI 211-1		CML. additive 0.1%	SEM	p-value

		0.1%	0.3%			
0 to 4 wk						
Avg. egg weight (g)	61.4	60.7	61.0	61.4	1.28	0.98
Egg breaking strength (kg/cm^2^)	3.93	3.78	4.14	3.70	0.21	0.40
Shell weight, % (shell weight/egg weight)	12.9	12.6	13.1	12.6	0.27	0.39
Shell thickness (μm)	0.40	0.43	0.42	0.44	0.02	0.57
Egg albumen height (mm)	6.81	6.87	6.88	6.48	0.32	0.51
Haugh unit	82.3	82.4	79.7	78.5	2.02	0.44
Yolk weight, % (yolk weight/egg weight)	27.2	28.4	28.8	28.5	0.61	0.30
Egg albumen weight, % (egg albumen weight/egg weight)	59.9	59.190	58.2	58.1	0.69	0.37
Yolk height (mm)	16.0	16.2	16.1	16.0	0.31	0.97
Yolk diameter (mm)	43.5	44.4	42.8	45.1	0.86	0.21
Yolk color, Roche scale	5.38	5.43	5.50	5.56	0.17	0.70
5 to 8 wk						
Avg. egg weight (g)	61.6	62.0	63.3	61.4	1.42	0.41
Egg breaking strength (kg/cm^2^)	3.95	3.93	3.94	4.05	0.21	0.68
Shell weight, % (shell weight/egg weight)	13.3	13.0	13.0	13.6	0.28	0.27
Shell thickness (μm)	0.42	0.44	0.46	0.42	0.02	0.34
Egg albumen height (mm)	6.62	6.48	6.47	6.52	0.36	0.87
Haugh unit	80.9	79.3	79.3	78.9	2.33	0.72
Yolk weight, % (yolk weight/egg weight)	26.8	29.0	27.9	29.5	0.69	0.26
Egg albumen weight, % (egg albumen weight/egg weight)	60.3^a^	58.4^ab^	59.2^ab^	57.9^b^	0.79	<0.05
Yolk height (mm)	15.9	16.3	16. 5	16.2	0.31	0.82
Yolk diameter (mm)	43.3	44.1	44.7	43.3	0.69	0.41
Yolk color, Roche scale	5.50	5.58	5.83	5.69	0.15	0.46
0 to 8 wk						
Avg. egg weight (g)	61.5	61.9	63.3	61.4	0.94	0.58
Egg breaking strength (kg/cm^2^)	3.93	3.79	4.03	3.86	0.15	0.51
Shell weight, % (shell weight/egg weight)	12.9	13.0	13.0	13.0	0.20	0.37
Shell thickness (μm)	0.41	0.43	0.44	0.42	0.02	0.53
Egg albumen height (mm)	6.79	6.67	6.57	6.48	0.23	0.52
Haugh unit	81.2	81.0	80.1	79.9	1.52	0.39
Yolk weight, % (yolk weight/egg weight)	27.0^b^	28.3^a^	28.3^a^	28.5^a^	0.45	<0.05
Egg albumen weight, % (egg albumen weight/egg weight)	60.1^a^	58.7^ab^	58.7^ab^	58.4^b^	0.45	<0.05
Yolk height (mm)	15.9	16.3	16.3	16.1	0.21	0.61
Yolk diameter (mm)	43.4	44.6	43.6	44.2	0.56	0.32
Yolk color, Roche scale	5.60	5.66	5.66	5.56	0.11	0.50

n = 12.

CML. additive: commercial *B. amyloliquefaciens* product; SEM, standard error of the mean.

a,b Means with different letters within the same row are significantly different at 5% level (p<0.05).

**Table 5:** Effects of dietary supplementation of *Bacillus subtillis* and *B. amyloliquefaciens* on egg quality of Leghorn laying hens during storage

Items	Control	TLRI 211-1		CML. additive 0.1%	SEM	p-value

		0.1%	0.3%			
Storage for 2 weeks						
Avg. egg weight (g)	59.7	60.0	61.0	59.8	1.18	0.41
Egg breaking strength (kg/cm^2^)	3.33^b^	4.23^a^	3.63^ab^	3.25^b^	0.22	<0.05
Shell weight, % (shell weight/egg weight)	11.9^b^	12.4^ab^	12.8^a^	12.9^a^	0.29	<0.05
Shell thickness (μm)	0.40	0.47	0.42	0.42	0.02	0.34
Egg albumen height (mm)	3.34	3.23	3.32	3.44	0.21	0.87
Haugh unit	70.2	68.3	68.4	70.1	3.27	0.72
Yolk weight, % (yolk weight/egg weight)	30.7	31.8	31.4	31.1	0.86	0.28
Egg albumen weight, % (egg albumen weight/egg weight)	57.5	55.6	56.2	56.1	0.98	0.14
Yolk height (mm)	10.0	10.5	10.6	10.6	0.31	0.37
Yolk diameter (mm)	50.9^ab^	50.1^b^	51.1^a^	51.6^a^	0.47	<0.05
Yolk color, Roche scale	6.10	6.35	6.39	6.33	0.17	0.46
Storage for 3 weeks						
Avg. egg weight (g)	57.7	60.2	58.2	60.3	1.42	0.84
Egg breaking strength (kg/cm^2^)	3.52	3.63	3.83	3.74	0.23	0.12
Shell weight, % (shell weight/egg weight)	12.5	12.5	13.1	13.1	0.28	0.82
Shell thickness (μm)	0.41	0.42	0.44	0.41	0.02	0.30
Egg albumen height (mm)	3.80^b^	4.76^a^	4.33^a^	3.84^b^	0.57	<0.05
Haugh unit	48.6^b^	56.2^ab^	60.3^a^	48.8^b^	4.18	<0.05
Yolk weight, % (yolk weight/egg weight)	31.4^b^	32.9^a^	34.8^a^	32.5^ab^	0.81	<0.05
Egg albumen weight, % (egg albumen weight/egg weight)	56.3	50.4	51.9	54.5	2.54	0.34
Yolk height (mm)	8.52	8.51	6.90	7.71	0.58	0.16
Yolk diameter (mm)	47.8^b^	56.6^a^	54.2^ab^	58.1^a^	2.78	<0.05
Yolk color, Roche scale	6.18^b^	6.45^a^	6.83^a^	6.08^b^	0.17	<0.05
Storage for 4 weeks						
Avg. egg weight (g)	55.4	55.8	57.8	55.3	1.15	0.54
Egg breaking strength (kg/cm^2^)	3.64	3.86	4.22	3.95	0.27	0.78
Shell weight, % (shell weight/egg weight)	13.0	13.2	12.7	12.9	0.27	0.17
Shell thickness (μm)	0.42	0.44	0.41	0.45	0.02	0.82
Egg albumen height (mm)	3.97	3.98	4.43	4.36	0.61	0.12
Haugh unit	43.0^b^	58.5^ab^	61.8^a^	60.3^a^	3.72	<0.05
Yolk weight, % (yolk weight/egg weight)	34.6	34.8	33.8	35.6	0.95	0.08
Egg albumen weight, % (egg albumen weight/egg weight)	51.9^b^	52.2^ab^	54.4^a^	51.5^b^	0.93	<0.05
Yolk height (mm)	7.84	7.12	7.77	7.48	0.51	0.16
Yolk diameter (mm)	56.6	56.1	55.4	60.4	4.01	0.15
Yolk color, Roche scale	6.22^b^	6.72^ab^	8.89^a^	6.57^ab^	0.17	<0.05

n = 12.

CML. additive: commercial *B. amyloliquefaciens* product; SEM, standard error of the mean.

a,b Means with different letters within the same row are significantly different at 5% level (p<0.05).

**Table 6:** Effects of dietary supplementation of *Bacillus subtillis* and *B. amyloliquefaciens* on blood characteristics of Leghorn laying hens^1)^

Items	Control	TLRI 211-1		CML. additive 0.1%	SEM	p-value

		0.1%	0.3%			
Glucose (mg/dL)	210	226	218	219	2.68	0.29
Amylase (U/L)	383	370	362	334	26.17	0.43
Triglycerides (mg/dL)	2,328^a^	2,553^a^	2,163^b^	3,332^a^	296	<0.05
Total cholesterol (mg/dL)	158	166	172	198	12.12	0.11
High density lipoprotein (mg/dL)	18	19	23	21	12.98	0.53
Low density lipoprotein (mg/dL)	12.4	12.04	13.3	15.5	2.39	0.12
Total bilirubin (mg/dL)	0.01	0.01	0.01	0.01	0.01	0.77
Glutamate oxaloacetate transaminase (U/L)	179	155	163	143	12.92	0.26
Glutamic pyruvate transaminase (U/L)	4.16	4.00	4.03	3.58	0.63	0.31
Alkaline phosphatase (U/L)	742	776	1,050	767	112	0.18
Creatinine phosphate kinase (U/L)	1,156	905	2,091	792	513	0.27
Uric acid (mg/dL)	3.72	4.24	4.40	3.93	0.37	0.56
Creatinine (mg/dL)	0.58	0.60	0.62	0.83	0.02	0.48
Urea nitrogen (mg/dL)	4.27	2.91	2.91	3.25	0.61	0.36
Total protein (g/dL)	5.42	5.52	5.38	5.59	0.14	0.73
Albumen (g/dL)	2.15	2.18	2.23	2.18	0.06	0.82
Globulin (g/dL)	3.27	3.34	3.15	3.38	0.11	0.40
Albumen/globulin ratio	0.66	0.65	0.72	0.65	0.02	0.23
Sodium (meq/L)	144^a^	143^a^	144^a^	140^b^	1.24	<0.05
Potassium (meq/L)	4.52	4.59	4.56	4.26	0.17	0.58
Calcium (mg/dL)	30.2^b^	31.5^ab^	33.1^a^	35.3^a^	1.45	<0.05
Phosphorous (mg/dL)	4.95	5.76	6.09	6.07	0.43	0.24

1) Average of the determination values at week 4 and 8, n = 16.

CML. additive: commercial *B. amyloliquefaciens* product; SEM, standard error of the mean.

a,b Means with different letters within the same row are significantly different at 5% level (p<0.05).

**Table 7:** Effects of dietary supplementation of *Bacillus subtillis* and *B. amyloliquefaciens* on blood immunoglobulin of Leghorn laying hens

Items	Control	TLRI 211-1		CML. additive 0.1%	SEM	p-value

		0.1%	0.3%			
4th weeks	----------------------------------------------------- μg/mL×10^3^ -----------------------------------------------------					
IgA	1.18^b^	1.14^b^	1.49^a^	1.48^a^	0.13	<0.05
IgM	7.81	8.32	8.08	8.66	0.52	0.24
IgG	213	231	218	273	22.34	0.23
8th weeks						
IgA	1.47	1.37	1.38	1.15	0.25	0.11
IgM	21.1	23.5	19.2	19.5	3.75	0.06
IgG	279	283	267	237	18.31	0.22
Means						
IgA	1.31	1.25	1.44	1.32	0.26	0.31
IgM	15.1	15.9	13.6	14.1	2.48	0.08
IgG	246	257	243	255	16.34	0.09

n = 8.

CML. additive: commercial *B. amyloliquefaciens* product; SEM, standard error of the mean; Ig, immunoglobulin.

a,b Means with different letters within the same row are significantly different at 5% level (p<0.05).
